# To the Deans of the Dental Colleges in the United States of North America

**Published:** 1884-08

**Authors:** Adolf Petermann

**Affiliations:** Frankfort-on-the-Main, Germany


### TO THE DEANS OF THE DENTAL COLLEGES IN THE UNITED STATES OF NORTH AMERICA.

*Deutsche Monatsschrift fuer Zahnheilkunde. Verlag von Arthur Felix, Leipzig.*

*Most Esteemed Gentlemen:*

I learn that representatives of all the dental colleges are to meet in New York on August 4th, for the purpose of discussing uniform rules of graduation.

The warm interest which I have always borne toward dental colleges causes me, most esteemed gentlemen, to address to you these lines, and to call your attention to abuses which I most urgently beg you to remedy. The above-mentioned meeting in New York appears to be especially appropriate to discuss the subject of my certainly not unfounded solicitation, and to bring about the *very necessary rapid redress.*

There is before me an article published in the INDEPENDENT PRACTITIONER, 1884, page 241, from the pen of *J. L. Tierney, D. D. S.*, an American. In this article these same abuses are discussed with the greatest objectiveness. Complaint is made in it that the dental colleges graduate *foreigners* (especially Germans) who do not possess even the slightest scientific preparation, and who attend college only from four to five months, in most cases without sufficient knowledge of the English language. The consequence of this is said to be, that men who in Germany but a short time previously had occupied the lowest positions as "barbers, corn operators, assistant dental mechanics," etc., return as D. D. S. after so short a sojourn in the United States.

I am sorry to be obliged to corroborate these statements of your countryman. Please permit me to mention only one of the numerous cases known to me, one of recent occurrence.

In September of last year a *barber* of this city, who had also been employed but a short time previously as independent dental operator, went to the United States of North America and entered a dental college much frequented by Germans. He attended English lectures from November until February, and ——— graduated as D. D. S. But in spite of this the man can scarcely read a few words in English, to say nothing of being able to follow an English lecture.

A large number of such men are already practicing in the German Empire as "American Doctors of Dental Surgery," graduated by *legally incorporated* colleges. At a *German* University these men would never have been admitted to a study — not to speak of an official dental examination — because it is simply unreasonable that anybody with so defective an education could successfully follow a study. How is it possible that *the very same men* who can scarcely master their mother-tongue are able to pass examination *with success* as D. D. S. at the American dental colleges, after having attended the *English* lectures from four to five months? How is it possible that these *foreigners* can acquire in so short a time that knowledge and fitness which *Americans by birth* can only attain after a two years' course? I have indeed to agree with my colleague, *Dr. Tierney*, when he says in the above mentioned article, that these "five-months' doctors," though not in form, yet in principle, are to be classed with those who obtained their diplomas from a *Buchanan* or from the *Wisconsin* college.

You will not be surprised, most esteemed gentlemen, that the dentists graduated in Germany do not consider as "colleagues" those *uneducated* persons who graduated in your country, and of whom I have just spoken. But what opinion of the American dental colleges must be entertained by the German public, if these same persons who five months ago were *barbers*, etc., appear now as "doctors of dental surgery?" It is only too evident that, unfortunately, under such conditions the innocent ones—and there are here in Europe certainly a great many excellent men among the American dentists—must suffer with the culprits.

Throughout the world, and not the least in my own country, have been gratefully acknowledged the high services for which dental art is indebted to the United States of North America. During a long time your dental colleges were a high school for dentists, and it could be justly said that the dentist who, in addition to his home university, had successfully attended an American college, had done all that possibly could be done for his dental education.

Formerly, therefore, many *German dentists*, after having acquired as thorough a knowledge as possible at home, would leave for the United States to perfect their studies at one of the dental colleges. To-day things are different. To-day it is mostly a number of *totally illiterate* men who, in the most favorable cases occupied themselves for some time as dental mechanics, flock to the American dental colleges, not with the intention to get a thorough training, but to obtain as quickly as possible the diploma as D. D. S. Through this abuse the formerly good reputation of your dental colleges has already suffered heavily, and will suffer even more in future if it is not speedily checked.

The *educated German dentists*, who might desire to enrich their experience at one of your dental colleges, would refrain from so doing by noticing with whom they would be obliged to associate at the college, and what incapable men they would be obliged to accept afterward as "colleagues."

I believe, most esteemed gentlemen and colleagues, that I have given you in a few traces a true picture of the circumstances which have already damaged the formerly good reputation of your dental colleges, and which threatens to destroy it in the future. As you, most esteemed gentlemen, are presumably aware of the interest that I have always taken in the American dental colleges, I take the liberty to beg you most humbly to direct your attention to the abuses depicted by *Dr. Tierney* and by myself, and to devise measures of speedy relief. Be careful, most esteemed gentlemen, that only actually efficient men will graduate in future from your dental colleges, and that to all those foreigners who cannot pass examination at home, or who, at least, have not attended there some university for a sufficient time, the degree of D. D. S. will only be conferred in case, after *several years* of study, they give an efficient proof of their fitness by a *thorough* examination.

It is only by strict adherence to some such rules that your dental colleges will regain their former standing with us in Germany.

With highest esteem,

Dr. Adolf Petermann,

Frankfort-on-the-Main, Germany.

